# Healthcare-associated hepatitis B and C transmission to patients in the EU/EEA and UK: a systematic review of reported outbreaks between 2006 and 2021

**DOI:** 10.1186/s12889-022-14726-0

**Published:** 2022-12-03

**Authors:** Jasleen Singh, Savina Stoitsova, Karolina Zakrzewska, Lukasz Henszel, Magdalena Rosińska, Erika Duffell

**Affiliations:** 1 European Centre for Disease Prevention and Control, Stockholm, Sweden; 2 European Programme for Intervention Epidemiology Training, European Centre for Disease Prevention and Control, Stockholm, Sweden; 3 National Institute of Public Health NIH, National Research Institute, Warsaw, Poland

**Keywords:** Hepatitis B, Hepatitis C, Healthcare-associated, Transmission route

## Abstract

Healthcare-associated transmission was the second most common hepatitis B (HBV) and hepatitis C (HCV) transmission route according to 2006–2012 European surveillance data, but data quality and completeness issues hinder comprehensive characterisation of this important issue. We carried out a systematic review of published literature on healthcare-associated transmission of HBV or HCV in European Union (EU) and European Economic Area (EEA) countries and the United Kingdom to complement surveillance data and identify higher-risk settings. We searched the PubMed and Embase databases and grey literature over the period January 2006 to September 2021, for publications reporting transmission events after 2000 in the EU/EEA and UK related to a healthcare setting or procedure. We collected data on the country, number of patients, setting type and route of transmission. In 65 publications from 16 countries, 43 HBV and 48 HCV events were identified resulting in 442 newly infected patients. Most events were reported from Italy (7 HBV and 12 HCV), Germany (8 HBV and 5 HCV) and the United Kingdom (8 HBV and 5 HCV). The number of patients infected from a single source within an event ranged from 1 to 53. Five large outbreaks of over 20 cases were identified, including two in Poland and one each in Belgium, Hungary and Slovakia. The majority of transmission events occurred through blood transfusions or in dialysis units. However, there were a number of outbreaks in seemingly low risk settings such as CT/MRI scanning units. A failure to adequately follow infection prevention control (IPC) precautions was reported in 30% of included studies. Healthcare-associated transmission of hepatitis B and C continues to occur in a range of community and hospital settings across EU/EEA countries and often results in large outbreaks, although the true extent of the situation cannot be fully determined due to under-reporting. Strict IPC precautions should be implemented across all healthcare settings and regularly audited, and surveillance systems strengthened and standardised to allow for comprehensive and consistent reporting of nosocomial transmission of hepatitis across the EU.

**Supplementary Information:**

The online version contains supplementary material available at 10.1186/s12889-022-14726-0.

## Introduction

In 2016, it was estimated that in the European Union (EU) and European Economic Area (EEA) and the United Kingdom (UK) there were approximately 4.7 million people living with chronic hepatitis B (HBV) and 3.9 million with chronic hepatitis C (HCV) [1]. HBV and HCV are bloodborne viruses (BBVs) and transmission can occur through exposure to contaminated bodily fluids. Healthcare-associated (nosocomial) transmission is the second most common HBV and HCV transmission route in routine surveillance data reported to the European Centre for Disease Prevention and Control (ECDC) by EU/EEA countries. In 2020, healthcare-associated transmission was responsible for 14% of acute HBV cases, and 23% of newly diagnosed HCV cases reported by EU/EEA countries among the cases for whom data were available [2, 3]. The long incubation times and difficulties differentiating between acute and chronic infection, especially for HCV, make it challenging to determine the exact transmission route. In addition, this information is captured only for a subset of cases in surveillance data and not reported by all countries. Nevertheless, the data highlight the importance of the issue and the need to further understand what drives transmission in order to support evidence-based measures to prevent future infections. Valuable insights on transmission mechanisms could be gained through analysing information from outbreak investigations. Whilst outbreaks are often presented at scientific conferences and in the medical literatures, there is no formal system in place to routinely collect information about nosocomial hepatitis outbreaks in the EU, unlike in the USA where this is collected annually by the Centers for Disease Control and Prevention (CDC) [4].

The European Network for Hepatitis B and C Surveillance [5] in 2015 recommended that a systematic review of published events of healthcare-associated transmission of hepatitis B and C in countries be carried out, in order to complement the surveillance data and bring about a better understanding of the determinants behind healthcare-associated transmission of HBV/HCV. While previous reviews of healthcare-associated transmission have been conducted [6, 7], there have been no recent reviews conducted focused on EU/EEA countries.

We conducted a systematic review with the objectives to describe events of healthcare-associated HBV/HCV transmission in the EU/EEA and UK in the published literature between 2006 and 2021, and to summarise the specific exposures and risk factors implicated in transmission.

## Methods

We focused on published reports of healthcare associated transmission events after 2000, where an event was defined as transmission of HBV/HCV to patients from a single source.

Using the PRISMA guidelines for systematic reviews [8] we formulated our research questions as:

1. What are the number of healthcare-associated transmission events of hepatitis B/C in the EU/EEA and UK, described in literature and in grey literature (including conference abstracts and technical reports) since 2006, per country, type of healthcare facility/service, and year (where event is defined as a cluster or outbreak, involving transmission to patients from a single source);
2. What are the specific exposures, identified as associated with the events of healthcare-associated transmission of hepatitis B/C, reported in literature (where specific exposure is defined as intervention or risk factor implicated in transmission).

These research questions implicate different outcomes (i.e. number of events, patients, types of facilities, specific exposures) within the population of patients attending healthcare settings and people using community healthcare services in any/all age groups. We considered any exposure to HBV or HCV in a healthcare setting or related to provision of healthcare services including inpatient and outpatient facilities, long-term healthcare facilities, community healthcare services and other specialised healthcare facilities, and related to healthcare procedures within these settings.

Following the PRISMA guidelines we combined concepts describing the population, exposure and outcomes, into a search strategy. Natural and controlled (MeSH/Emtree) vocabulary was used, involving terms that describe three concepts: hepatitis B/C (11 terms), patients (48 terms) and transmission event (8 terms). The strategy was first developed for PubMed, where it was checked for sensitivity against a list of previously identified eligible articles, and then revised accordingly. It was further adapted to Embase, by checking the meaning and position in the term tree of each MeSH term and ensuring concordance with the corresponding Emtree terms (see Appendix 1 for further details).

**Fig. 1:**
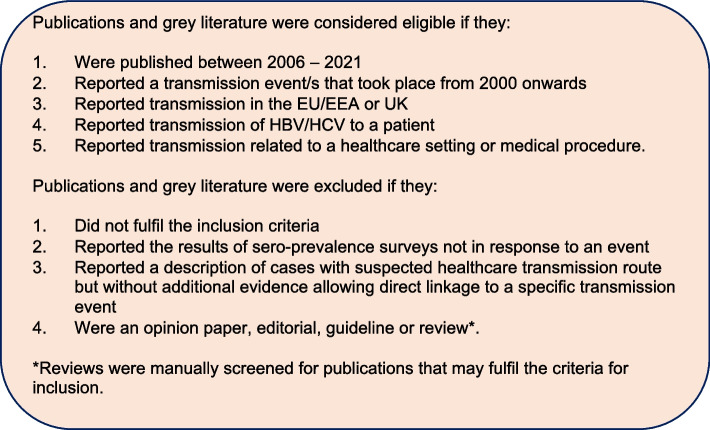
Inclusion and exclusion criteria

Using this strategy, in October 2021 we searched PubMed and Embase databases (January 2006 to September 2021) for publications reporting transmission events after 2000. Publications and grey literature, including conference abstracts and other relevant articles (such as court cases) in all languages were included and translated using online translation. We also searched the references of relevant reviews included in our search, and identified candidate records from the Outbreak Database (a worldwide database for nosocomial outbreaks supported by the Charité – University Medicine Berlin^1^). Full inclusion and exclusion criteria used in the screening of identified records are detailed in Fig. 1. References were managed and exported using Endnote.

Four people carried out the abstract and title screening and two carried out the full text screening and data extraction. At the beginning of each screening step a subset of publications was screened in parallel by all team members and decisions were compared until 95% congruence in decisions was reached, at which point team members continued to screen independently. Ongoing discussion with the project team took place throughout the screening and data extraction process to guide the process and ensure criteria were applied uniformly. Although this deviates from standard practise of two researchers conducting screening and data extraction, this method of reviewing nevertheless ensured a consistent application of the inclusion criteria.

We collected data on country, publication year, event/transmission year (year transmission took place; if event spanned > 1 year—the first year of transmission), number of patients (number of patients infected excluding source patient; if source patient was not clearly identified among patients—total number of patients), healthcare setting, specific exposure(s) implicated and study methodologies used. If an event was reported in two articles, it was treated as one event. Results were collated and simple descriptive analysis to summarise the findings was undertaken. The results of this analysis are presented in basic figures produced using Excel and using the ECDC Map Maker tool EMMa [9]. Full details of the data collection process are outlined in Appendix 2. 

Included studies were divided into three study types: lookback investigations; studies reporting phylogenetic analysis; and studies not reporting phylogenetic analysis. Phylogenetic analysis and whole genome sequencing is the gold standard for epidemiological outbreak investigations, facilitating identification of the transmission pathway [10]. Lookback investigations are a specific design of study usually used in transfusion medicine to identify and notify recipients who may have received blood products from an infected donor [11]. No risk of bias was undertaken for the studies in view of the heterogeneity of the articles.

## Results

**Fig. 2:**
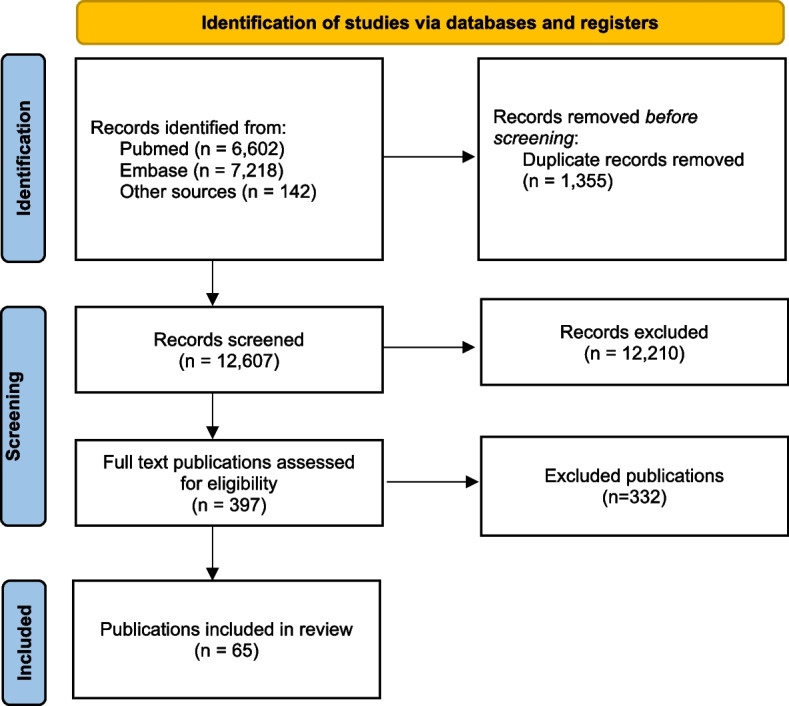
PRISMA flowchart

The search of PubMed and Embase databases for the period 2006–2021 resulted in a total of 13,818 publications. An additional 142 publications were identified from other sources (including 51 snowball references and 91 records from the Outbreak Database). After the title/abstract screen, 397 publications were included for full text screen, and 65 were included for data extraction (Fig. 2). The included articles provided information on 91 outbreak events from 16 countries, involving a total of 442 cases and 25 fatalities, 5 of which were related to hepatitis. A total of 48 (53%) outbreaks were related to hepatitis C and 43 (47%) to hepatitis B. A summary of all included outbreak events is reported in Appendix 3.

According to the date when the events occurred, there were a median of five events reported in the literature each year (range 1–14), with a median of five years (range 0–14) between year of event and year of publication. The latest event was in 2016. Taking into account publication delay, the number of events remained stable over time.

**Fig. 3:**
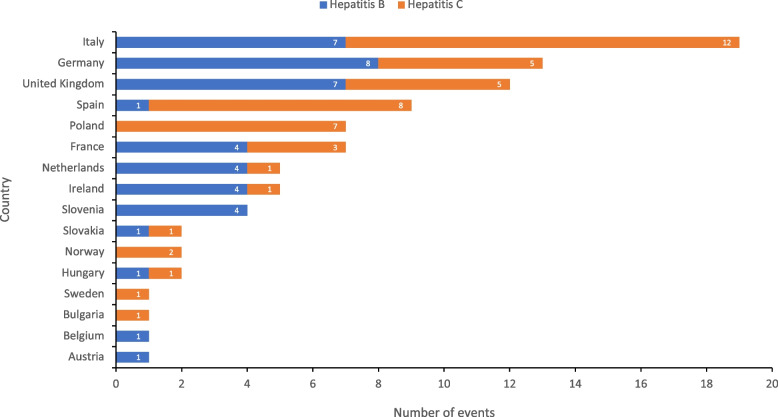
Results from literature review: number of healthcare associated HBV/HCV events reported in the EU/EEA and UK, 2006–2021

**Fig. 4:**
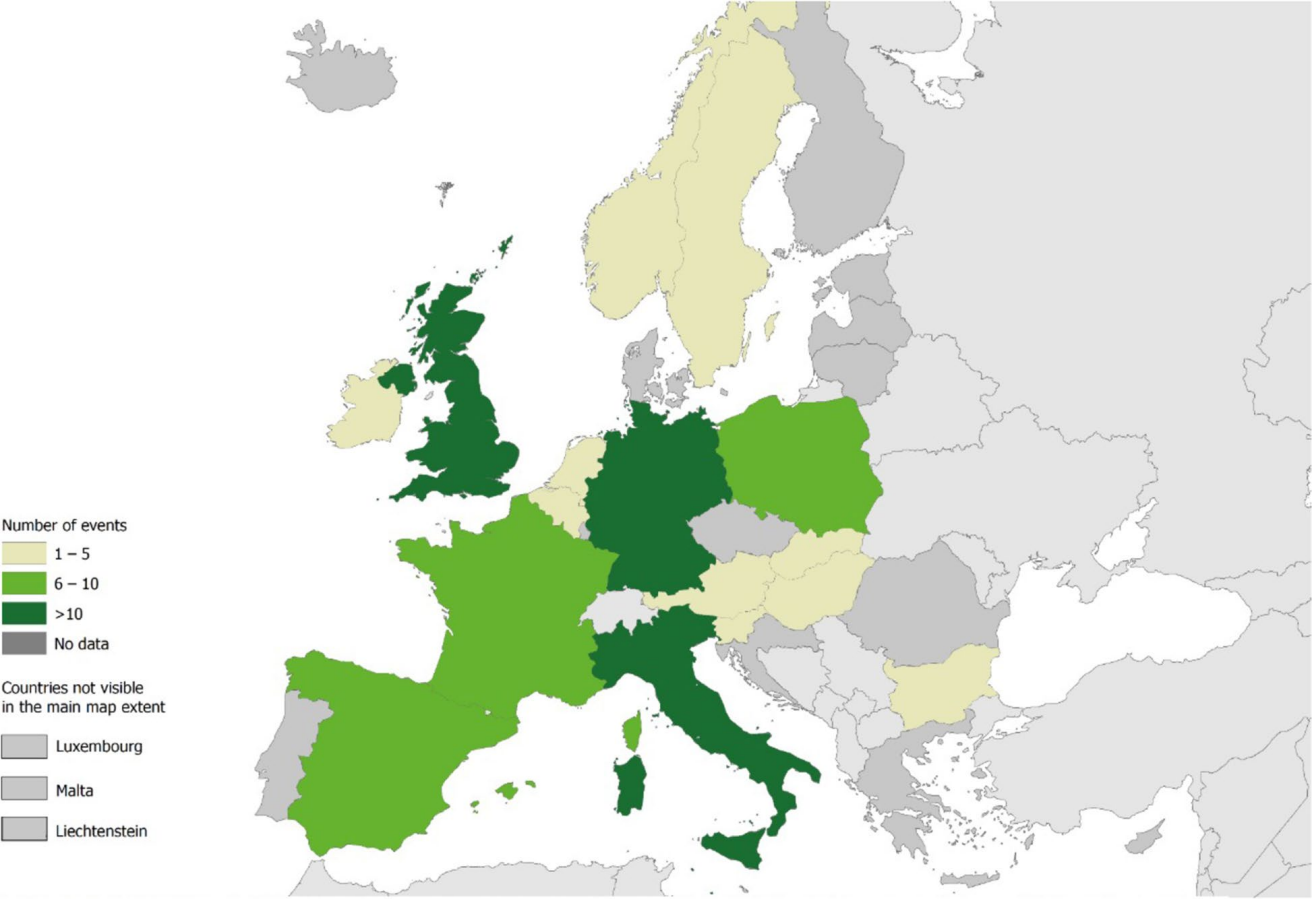
Results from literature review: map of healthcare associated HBV/HCV events reported in the EU/EEA and UK, 2006–2021. EEA – European Economic Area, EU – European Union, HBV – hepatitis B virus, HCV – hepatitis C virus, UK – United Kingdom

The majority of events published in the literature were from Italy (21%, 7 HBV and 12 HCV), Germany (14%, 8 HBV and 5 HCV), the UK (13%, 8 HBV and 5 HCV) and Spain (10%, 1 HBV and 8 HCV) (Figs. 3 and 4). The number of identified patients infected from a single source within an event ranged from 1 to 53. Large outbreaks of > 20 cases were reported from Poland (2 outbreaks), Belgium, Hungary and Slovakia (1 outbreak each). Among countries from which > 3 transmission events were published, median event size (median number of cases per transmission event) ranged from 1 (France, Ireland and the United Kingdom) to 8 (Poland).

**Fig. 5:**
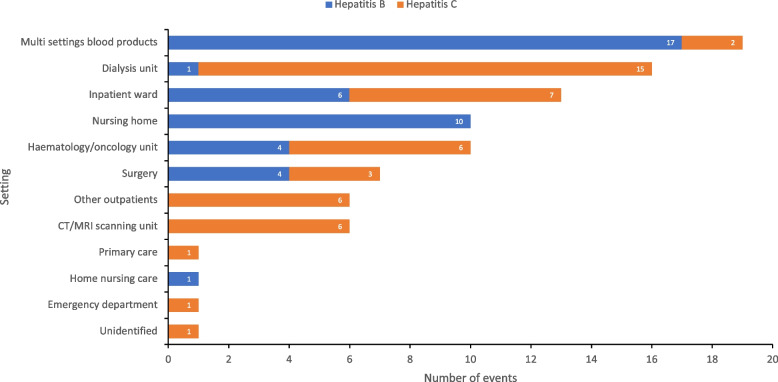
Number of healthcare associated HBV/HCV events by setting type reported in the EU/EEA and UK, 2006–2021. CT – computed tomography, EEA – European Economic Area, EU – European Union, HBV – hepatitis B virus, HCV – hepatitis C virus, MRI – magnetic resonance imaging, UK – United Kingdom

As shown in Fig. 5, the most common settings reported included ‘multi settings’ for events involving blood products (where no specific setting was identified) (21%, 17 HBV and 2 HCV), dialysis units (18%, 1 HBV and 15 HCV), inpatient wards (14%, 6 HBV and 7HCV), nursing homes (11%, 10 HBV) and haematology/oncology units (11%, 4 HBV and 6 HCV). Outbreaks in inpatient settings accounted for 49% of reported events with 21% reported from outpatient settings (inpatient settings were considered those where the patient is admitted to hospital, and outpatient were any other settings where care was given without requiring hospitalisation). Out of the 16 reported events in dialysis units, eight were specifically attributed to infection prevention control (IPC) breaches and three to the haemodialysis machine itself, with the remaining five unidentified. Of these 16 outbreaks, 15 involved HCV transmission and one HBV.

For each of the settings described above, the average number of cases per event setting was assessed and this indicated that the largest average number of cases was reported in primary care (32 cases from one event) followed by haematology/oncology units (9.3 cases), nursing homes (6.2 cases), CT/MRI scanning units (6 cases) and dialysis units (5.8 cases). A total of 6 events identified transmission from infected staff to patients, with 4 events occurring during surgery, 1 in an inpatient ward and 1 in primary care.

**Fig. 6:**
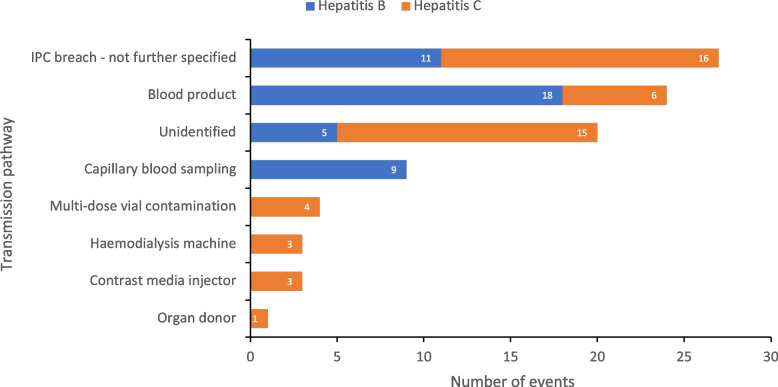
Number of healthcare associated HBV/HCV events by transmission pathway in the EU/EEA and UK, 2006–2021. EEA – European Economic Area, EU – European Union, HBV – hepatitis B virus, HCV – hepatitis C virus, IPC – infection prevention control, UK – United Kingdom

The transmission pathway was reported for 78% of events (Fig. 6). The most common factor identified was ‘IPC breach – not further specified’ (30%, 11 HBV and 16 HCV), followed by blood products (26%, 18 HBV and 6 HCV), capillary blood sampling (10%, 9 HBV), multi-dose vial contamination (4%, 4 HCV), haemodialysis machine (3%, 3 HCV), contrast media injector (3%, 3 HCV) and organ donors (1%, 1 HCV). Capillary blood sampling was implicated in all nursing home outbreaks, except two where the transmission pathway was unidentified.

**Fig. 7:**
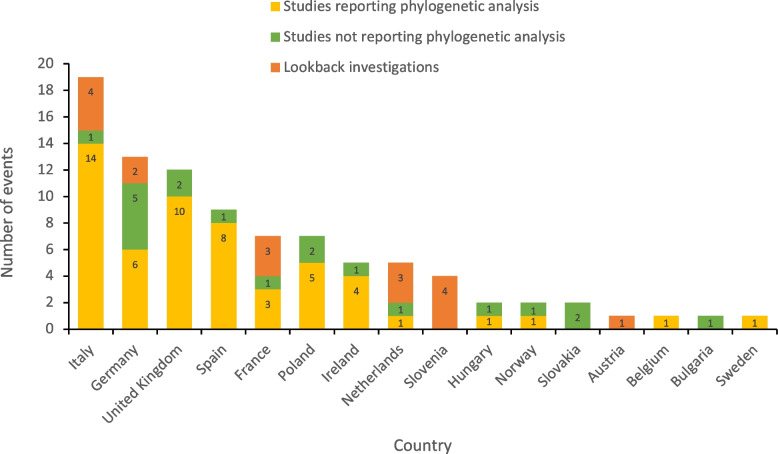
Number of healthcare associated HBV/HCV events by study type and country in the EU/EEA and UK, 2006–2021. EEA – European Economic Area, EU – European Union, HBV – hepatitis B virus, HCV – hepatitis C virus, UK – United Kingdom

Considering events by study type and country (Fig. 7), countries reporting the highest number of lookback investigations were Italy and Slovenia (4 events each), followed by France and the Netherlands (3 events each). Countries with the highest number of events reporting phylogenetic analysis were Italy (14 events), the UK (10 events) and Spain (8 events).

## Discussion

The results from this systematic review provide an overview of published studies focused on nosocomial hepatitis transmission in EU/EEA countries and the UK to complement data from newly diagnosed cases reported through surveillance. We found 91 transmission events reported in the published medical literature from 16 EU/EEA countries and the UK over a 15 year period from 2006 to 2021. A range of setting types were implicated with most events occurring through blood transfusions or in dialysis units. A number of outbreaks occurred in seemingly low risk settings such as CT/MRI scanning units.

Outbreaks in inpatient settings accounted for half of all reported events. Inpatient settings tend to report a higher number of nosocomial infections than outpatients (where patients are not admitted overnight) which is likely due to various factors, including the complexity of healthcare delivery in inpatient settings, frequency of medical procedures and better monitoring and reporting systems in place [12].

Events classified as ‘multi setting blood products’, which includes all transfusion related events, accounted for a large proportion of outbreaks identified in published studies. This was included as a separate category as the majority of studies describing transfusion related infections did not specify a setting for transmission. Note that publications on studies of transmission through blood products use a look-back approach, where registries of past transfusions from identified infected donors are retrospectively screened. These specific studies allow for active case-finding over large periods of time. This means that the large proportion of transmission events compared to other settings is likely due to both publication bias and the high sensitivity of this type of investigation. Even as transmission events can still be identified related to blood transfusion, dedicated studies have demonstrated that blood transfusions have become significantly safer with the introduction of robust blood screening protocols [13, 14].

Consistent with findings from previous research [15], many outbreaks (18%) occurred in dialysis units and all except one were related to HCV. It is not clear why the outbreaks in dialysis units were mostly due to HCV, but this could be attributed to protection of these patients from HBV from the widespread rollout of immunisation [16]. However, the challenge of achieving good vaccine response in end-stage renal disease patients is well recognised [17] and similar hepatitis B and C incidence has been documented among dialysis patients in some settings [18]. Therefore, we cannot exclude some publication bias to account for our findings.

Events in dialysis units were reported from across many countries. Bloodborne infections are well recognised as an important hazard in dialysis units, and prevalence of HCV among dialysis patients is generally higher than the general population [19]. The interplay between dialysis and the risk of bloodborne infections is complex but the potential risk is great due to frequent vascular access, poor immune function and malnutrition among dialysis patients, as well as the inherent risks of prolonged inpatient stays [20].

The majority of reported outbreaks in dialysis units were attributed to breaches in standard IPC measures, such as cross-contamination from equipment or surfaces. IPC breaches included staff not consistently changing gloves and washing hands between patients, incomplete disinfection of surfaces, and unsafe injection practises such as the use of multidose vials [21–23]. There has been substantial progress to mitigate the risk of nosocomial outbreaks among dialysis units through the implementation of ‘universal’ or ‘standard’ precautions for preventing bloodborne virus (BBV) transmission [24] and reductions in prevalence have followed strict enforcement of IPC measures. For example, a prospective study of over 1700 haemodialysis units in Belgium found a reduction in HCV prevalence from 13.5% in 1991 to 6.8% in 2000, with a similar trend reported in five other European countries (France, Sweden, Italy, the UK and Hungary) [25]. Despite this, dialysis units remain a key setting for hepatitis transmission and strict enforcement of standard precautions, periodic audits of practice and regular staff training are all essential.

The need for strengthening IPC procedures and staff training is also evidenced by occasional outbreaks in other inpatients settings, as outbreaks occurred across multiple types of wards, as well as in nursing homes. Although the exact mechanism of transmission was sometimes unclear, investigations revealed various deficiencies such as the multiple use of disposable needles [23], surface contamination of shared equipment [25], or non-compliance with basic hand hygiene [25].

Infections related to surgery accounted for the highest number of events where transmission occurred from an infected healthcare worker (HCW) to a patient, with six outbreaks identified. This is consistent with other research showing that most outbreaks related to HCWs occur as a result of transmission during surgery [26]. One outbreak occurred in primary care, in which a primary care physician in Belgium unknowingly infected 32 patients with HBV through unsafe infection practises, demonstrating the extent of transmission that can occur from an infected HCW [27]. For HCV infected HCWs the guidance is clear as the treatment for those infected involves a straightforward regime of direct-acting antivirals (DAAs) to clear the infection [28], but policies for HBV positive HCWs varies among countries. The CDC recommends treating some HBV infected HCWs with antiviral therapy even if they do not fulfil the typical indications for treatment, to reduce the possibility of transmission during exposure prone procedures (EPPs) to patients [29]. Regular screening and vaccination of HCWs (particularly those performing EPPs) in combination with IPC training, is essential in order to avoid harm to patients as well as protect staff from occupationally acquired infections [30]. Finally, settings that are not often recognised as risky areas for nosocomial outbreaks were also identified, with 7% of outbreaks (six in total) occurring in CT/MRI scanning units. Half of these outbreaks related to the unsafe use of contrast media injections resulting in transmission between patients, and the other half were unidentified. Automatic injectors used for IV administration of contrast agents during CT/MRI scanning have previously been highlighted as an important IPC risk in radiology units [31]. Strict adherence to disinfection procedures as well as regular training on the use of automatic injectors is therefore needed to reduce the risk of contamination.

Whilst suboptimal practice in IPC accounted for the majority of transmission events, transmission through blood products was the second most commonly reported over the period of the study, with stable trends over time. Historically, transfusion associated hepatitis was a major cause of HBV and HCV infections; during the 1960’s the risk of acquiring hepatitis from a single transfusion was estimated at 30% [32]. Since then the risk in Europe has been drastically reduced by strict EU legislation mandating the use of risk based assessments for donors and screening of blood products, including viral nucleic acid testing (NAT) for HBV and HCV [33]. A particular benefit of NAT screening is the ability to detect occult HBV infection (OBI), defined as the presence of detectable HBV DNA in individuals who are HBV surface antigen (HBsAg) negative [34]. All EU/EEA countries now have robust haemovigilance systems in place and screen donations using quality assured methods [35]. This quality control of blood products has had a major impact on reducing the incidence of transfusion associated infections including hepatitis [36].

Nowadays the safety of blood products is related to the incidence of HBV and HCV with residual risk connected to transfusion of blood from a donor in the window period for NAT [37]. In addition there has been growing evidence of HBV transmission from donors with OBI [38, 39] although our review was not able to assess the extent of this issue among the events reported. OBI prevalence correlates with HBV prevalence in the population of blood donors; a 2017 systematic review found the prevalence of chronic HBV infection among first-time blood donors ranged from 0 to 3.2% across 30 European countries, and the prevalence of anti-HCV ranged from 0 to 2.2% [40]. This review found higher HBsAg and anti-HCV prevalence rates in the Eastern and Southern part of the EU/EEA compared to the West, indicating higher residual risk in Eastern and Southern parts of Europe. Conversely, we found that most blood product related transmission events were reported from Western European countries, although this may be explained by reporting and/or publication bias.

Capillary blood sampling (CBS) devices were implicated in one haematology/oncology unit event and in all nursing home events, except for two which could not identify the transmission pathway. Eight events involving CBS devices and the transmission of HBV were reported from just four countries (the UK, Germany, Italy and the Netherlands) between 2001 and 2007. Research suggests that HBV outbreaks in nursing homes are influenced by two conditions: the fact that HBV infections in the elderly are often asymptomatic, and that nursing homes are settings of regular glucose monitoring for diabetic residents, which inherently carries a risk of patient-to-patient transmission unless strict precautions are followed [41]. Transmission of BBVs through non-disposable multi-patient lancet devices has been extensively documented [42, 43], which has led to a shift towards the use of disposable lancets in healthcare settings for monitoring glucose levels of diabetic patients with clear guidance that fingerstick devices should never be used for more than one patient [44]. The articles included in this review are likely to underestimate the true scale of the problem, but reassuringly we found no articles recording CBS devices as the transmission mechanism after 2007, which perhaps reflects the increased awareness and widespread implementation of related guidance.

Lastly, use of multidose vials was identified as an important cause of transmission accounting for 4% of all events. One article from Italy reports an HCV outbreak involving two ward nurses on a haematology/oncology unit infecting five patients through the repeated use of multidose saline flushes during the rinsing of CVC’s (central venous catheters) [45]. Similarly, an outbreak in a thalassaemia outpatients department in Italy resulted in four clusters of HCV infections related to multidose vials [46]. Research suggests that HCV can be transferred via sterile needles and syringes into medication vials if the diaphragm is contaminated with enough quantity of HCV, and the virus remains stable in several commonly used medications [47]. In the 1990’s, nosocomial HCV outbreaks attributed to poor injection practises resulted in the US CDC to develop the ‘One and Only’ campaign, which advocated the use of ‘one syringe, one needle at one time’ [48]. Despite this, multidose vials continue to be widely used in certain specialties such as nuclear medicine, where they are needed to reduce the operator’s radiation exposure when making up vials of radioactive product [49]. The WHO has developed specific guidance on multidose vials in order to reduce unsafe injection practises associated with their use [50].

The countries reporting the highest number of events were Italy, Germany and the UK. There is some evidence of a geographical gradient with fewer outbreaks reported from countries in Eastern Europe compared to the West, and outbreaks reported from Eastern European countries were larger in size. Various factors may explain this finding such as a variation in local reporting and surveillance practises as well as differing practises around publishing events in the literature. In addition, differences in the underlying epidemiology of hepatitis across regions may also have an impact, with HBV and HCV prevalence highest among countries in the South and Eastern part of Europe [51].

The WHO acknowledges that most countries lack robust surveillance systems for detecting healthcare associated infections (HAI), and those that do often struggle with the lack of standardised criteria for diagnosing infections [52]. In 2004 the WHO Regional Office for Europe issued recommendations for countries to establish minimum standards of infection control and a surveillance system to monitor HAI through processes and outcome indicators [53]. Since then the ECDC has established HAI-NET, a European network for the surveillance of healthcare associated infections that coordinates the European point prevalence survey of HAI and antimicrobial use [54]. The future inclusion of hepatitis specific indicators as part of this surveillance could be considered to provide further information on the scale nosocomial transmission of hepatitis across the EU/EEA.

One of the key limitations of using a systematic literature review to obtain an overview of nosocomial transmission is the publication bias that limits comparisons between countries. Some countries have more robust surveillance systems and public health resources to identify, investigate and report nosocomial transmission events than others. Undoubtedly, published studies account for a small proportion of all detected outbreaks and in addition, the long incubation period and typically asymptomatic course of infection for acute HBV and HCV further complicate detection of outbreaks. The true burden of nosocomial transmission is clearly higher than documented by surveillance data reported to the ECDC, notwithstanding the incompleteness of these data. Importantly, we identified nosocomial outbreaks even in countries that are not able to report transmission routes for the surveillance data.

Differences between countries and outbreak settings are often difficult to interpret as included studies use different study designs, case ascertainment methods and even laboratory tests. In addition, outbreak investigations involving epidemiological techniques and phylogenetic analysis are the strongest sources of evidence included in this review. Studies reporting phylogenetic analysis made up the bulk of all included papers, followed by those not reporting phylogenetic analysis and finally lookback investigations. Phylogenetic analysis should be recommended, although resources for such investigations may not always be available which can make it challenging to confirm hepatitis B or C transmission events [55]. Look-backs are comprehensive investigations that demand a great deal of time and resources, which may explain why we only found these in a few countries. Due to the inclusive nature of our search which also captured conference abstracts, forensic investigations and court cases, our results were derived from a range of sources with data presented in a range of different formats. Due to this heterogeneity it was not feasible to apply a standardised risk of bias assessment tool across the included studies. This highlights the need for greater standardisation in the reporting of healthcare-associated infections in order to allow for valid comparisons between studies.

There is a clear need to encourage consistent reporting of nosocomial transmission events across the EU/EEA. Currently there is variability in surveillance practice and combined with insufficient data this limits meaningful comparisons across the region [56]. The development of clear indicators and standardised methodological approaches for the reporting of suspected healthcare related transmission events are needed. The Outbreak Database (a worldwide database for nosocomial outbreaks supported by the Charité—University Medicine Berlin) [57] is a source of information on published nosocomial outbreaks worldwide which could be better utilised by infection control specialists.

The majority of articles included in this review identified that breaches in infection prevention control practises were a contributing, if not the major, factor in nosocomial hepatitis events. Despite an abundance of guidance published on mitigating the risk for nosocomial transmission, deficiencies in following standard precautions exist across all settings types and countries. There is therefore a need to strengthen basic IPC procedures in all settings including in the community, with emphasis on regular staff training and audit. The importance of protecting HCWs through hepatitis B vaccination and regular screening for bloodborne viruses has also been highlighted in this review through the reporting of several outbreaks involving infected staff. Finally, there must be strengthened coordination between institutions and countries to share high-quality data and good practise, in order to enable a coordinated response to the challenge of nosocomial hepatitis.

## Conclusion

In summary, our review found evidence of ongoing transmission of HBV and HCV in healthcare settings across the EU/EEA and UK. The results of our study highlight diversity in reported nosocomial events in the published medical literature, with common elements such as a failure to follow IPC precautions prevalent across most reported studies. Events were reported from settings known to pose higher risk (dialysis units), but also from settings perceived to be at lower risk (CT/MRI scanning units), which underlines the importance of following universal precautions in all settings and possibly the need to carefully review procedures to enhance the implementation of universal precautions in settings like radiology units. Differences between countries exist but the results are likely to be strongly influenced by a publication bias. Collation and analysis of unpublished information on incidents from countries may improve understanding of the true extent of nosocomial transmission.

## Supplementary Information

**Additional file 1:** **Supplementary file 1.**

## Data Availability

Not applicable.

## Footnotes

1 https://www.outbreak-database.com/Home.aspx

## Abbreviations

CBS: Capillary Blood Sampling
CDC: Centers for Disease Control and Prevention
CT: Computed Tomography
CVC: Central Venous Catheter
DAA: Direct-Acting Antiviral
ECDC: European Centre for Disease Prevention and Control
EEA: European Economic Area
EPP: Exposure Prone Procedure
EU: European Union
HAI: Healthcare Associated Infection
HBV: Hepatitis B Virus
HBsAg: Hepatitis B Surface Antigen
HCV: Hepatitis C Virus
HCW: Healthcare Worker
IPC: Infection Prevention Control
MRI: Magnetic Resonance Imaging
NAT: Nucleic Acid Testing
OBI: Occult Hepatitis B Infection
UK: United Kingdom
WHO: World Health Organisation
