# Supplementary material for: Healthcare-associated hepatitis B and C transmission to patients in the EU/EEA and UK: a systematic review of reported outbreaks between 2006 and 2021
Source: BMC Public Health. 2022 Dec 3;22:2260. doi: 10.1186/s12889-022-14726-0 (PMC9719626; doi:10.1186/s12889-022-14726-0)
Supplement: Supplementary file 1 — Additional file 1: Supplementary file 1. [file 12889_2022_14726_MOESM1_ESM.docx]

**Appendices**

**Appendix 1: Search Strings**

**Pubmed Search String:**

(“Hepatitis B” OR HBV OR “Hepatitis C” OR HCV OR “Hepatitis B” [MeSH Terms] OR “Hepatitis B Antigens” [MeSH Terms] OR “Hepatitis B Antibodies” [MeSH Terms] OR “Hepatitis B virus” [MeSH Terms] OR “Hepatitis C” [MeSH Terms] OR “Hepatitis C Antibodies” [MeSH Terms] OR “Hepatitis C Antigens” [MeSH Terms])

**AND**

(Patient [Text Word] OR Patients [Text Word] OR Outpatient [Text Word] OR Outpatients [Text Word] OR Patients [MeSH Terms] OR “Homebound person” [Text Word] OR “Homebound persons” [Text Word] OR “Homebound persons” [MeSH Terms] OR “Disabled person” [Text Word] OR “Disabled persons” [Text Word] OR “Disabled persons” [MeSH Terms] OR ”Health facility” OR “Health facilities” OR “Health facilities” [MeSH Terms] OR Hospital [Text Word] OR Hospitals [Text Word] OR Hospitals [MeSH Terms] OR “Home care agency” [Text Word] OR “Home care agencies” [Text Word] OR “Home care agencies” [MeSH Terms] OR “Voluntary health agencies” [MeSH Terms] OR “Health personnel” [MeSH Terms] OR “Health service” [Text Word] OR “Health services” [Text Word] OR “Health services” [MeSH Terms] OR “Health care associated” OR “Healthcare associated” OR “Iatrogenic” OR “Hospital acquired” OR “Cross infection” OR Nosocomial OR “Iatrogenic disease” [MeSH Terms] OR “Healthcare associated infections” [Text Word] OR “Associated infection, healthcare” [Text Word] OR “Associated infections, healthcare” OR “Healthcare associated infection”[Text Word] OR “Infection, Healthcare Associated” [Text Word] OR “Infections, Healthcare Associated” [Text Word] OR “Infection, Cross” [Text Word] OR “Health care associated infections” [Text Word] OR “Health care associated infection” [Text Word] OR “Hospital infections” [Text Word] OR “Hospital infection” [Text Word] OR “Infection, hospital” [Text Word] OR “Infections, hospital” [Text Word] OR “Infection, nosocomial” [Text Word] OR “Infections, nosocomial” [Text Word] OR “Nosocomial infection” [Text Word] OR “Nosocomial Infections” [Text Word])

**AND**

(Outbreak [Text Word] OR Outbreaks [Text Word] OR “Disease Outbreaks” [MeSH Terms] OR “Cluster analysis” [MeSH Terms] OR “Cluster” OR “Clusters” OR Transmission [Text Word] OR “Disease transmission, Infectious” [MeSH Terms])

**Embase Search String**

‘hepatitis b’ OR hbv OR ‘hepatitis c’ OR hcv OR ‘hepatitis b’/exp OR ‘hepatitis b antigen’/exp OR ‘hepatitis b antibody’/exp OR ‘hepatitis b virus’/exp OR ‘hepatitis c’/exp OR ‘hepatitis c antibody’/exp OR ‘hepatitis c antigen’/exp OR ‘hepatitis c virus’/exp

**AND**

patient:ti,ab OR patients:ti,ab OR outpatient:ti,ab OR outpatients:ti,ab OR ‘homebound patient’:ti,ab OR ‘homebound patients’:ti,ab OR ‘disabled person’:ti,ab OR ‘disabled persons’:ti,ab OR ‘health care facility’:ti,ab OR ‘health care facilities’:ti,ab OR hospital:ti,ab OR hospitals:ti,ab OR ‘home care agency’:ti,ab OR ‘home care agencies’:ti,ab OR ‘home care’:ti,ab OR ‘health service’:ti,ab OR ‘health services’:ti,ab OR ‘health care associated infections’:ti,ab OR ‘cross infection’:ti,ab OR ‘hospital infection’:ti,ab OR ‘nosocomial infection’:ti,ab OR ‘health care facility’ OR ‘health care facilities’ OR ‘healthcare associated infection’ OR ‘hospital acquired’ OR ‘cross infection’ OR ‘nosocomial’ OR ‘nosocomial transmission’/exp OR ‘patient’/exp OR ‘disabled person’/de OR ‘mental disease’/de OR ‘mental deficiency’/de OR ‘health care facility’/mj OR ‘hospital’/mj OR ‘hospice’/exp OR ‘health service’/mj OR ‘health care personnel’/mj OR ‘iatrogenic disease’/de

**AND**

outbreak:ti,ab OR outbreaks:ti,ab OR transmission:ti,ab OR cluster OR clusters OR ‘disease outbreak’ OR ‘cluster analysis’/exp OR ‘disease transmission’/exp

**Appendix 2: Data collection process**

After linking multiple reports pertaining to the same study (by time/place/person), relevant data was extracted from each included article. A study is defined as a report/reports related to a single instance of healthcare-associated hepatitis B and C infection. One article may include more than one study.

A random selection of 5 of the first 100 articles was made, selected by each reviewer on the basis of title and abstract. The full text of each of these articles was then read by two reviewers and data extraction performed. If data extraction was consistent (less than 5% inconsistency), the reviewers continued data extraction separately (one reviewer per article). If not, the data extraction tool in **Table 1** was consulted and the process repeated. Data extraction was performed simultaneously with full text screening. The reviewers screened the full texts of selected references separately. Data from each study was extracted using a predefined set of variables (see **Table 1**).

**Table 1. Data extraction variables**

| **Variable** | **Description** | **Values** |
| --- | --- | --- |
| PMID or DOI | PubMed identifier, accession number or DOI | String / . for missing |
| Study ID | Unique study ID | Numerical – provided to reviewer with abstract |
| Study title | Title of article | String |
| Year of publication | Year of publication | Year: yyyy |
| Disease | Disease for which the study reports healthcare-associated hepatitis infection | Categorical, pre-labeled:  1.HBV  2.HCV |
| Country | Country for which the study reports healthcare-associated hepatitis infection | Country name |
| Type of study | Type of study (if not expressly defined by authors, use best judgement) | Categorical, pre-labelled:  1. Lookback investigations  2. Studies reporting phylogenetic analysis  3. Studies not reporting phylogenetic analysis |
| Year/s of diagnosis of patients in outbreak | Fill in year of diagnosis of patients in outbreak | yyyy OR yyyy-yyyy |
| Setting group | Setting group where event has occurred | Categorical, pre-labelled:  1. CT/MRI scanning unit  2. Dialysis unit  3. Emergency department  4. Haematology/oncology unit  5. Home nursing care  6. Inpatient ward  7. Multi setting blood products*  8. Nursing home  9. Outpatients  10. Primary care  11. Surgery  12. Unidentified  *‘Multi setting blood products’ encompasses all transfusion related events where no specific setting was identified. |
| Number of events | Number of nosocomial hepatitis events | Numeric |
| Number of cases | Number of cases | Numeric |
| Number of deaths | Number of deaths split by ‘hepatitis related’ and ‘non-hepatitis related’ | Numeric and categorical, pre-labelled:  1. Hepatitis related  2. Non-hepatitis related |
| Source | Source of transmission | Categorial, pre-labelled:  1. Blood product  2. Capillary blood sampling  3. Contrast media injector  4. Haemodialysis machine  5. Multi-dose vial contamination  6. Organ donor  7. Unidentified  8. IPC breach – not further specified*  *’IPC breach – not further specified’ refers to scenarios where an infection prevention and control breach was identified but could not be categorically linked to transmission. |
| Intervention | Intervention reported to be associated with transmission | String |
| Risk factor | Procedures/risk factors reported to be associated with transmission | String |
| Was genetic/phylogenetic analysis performed to ascertain linkage  among source/cases | Was genetic/phylogenetic analysis performed to ascertain linkage among source/cases | Yes/No |
| If genetic/phylogenetic analysis performed,  details of analysis | Enter details of methodology behind genetic/phylogenetic analysis | String |
| Comments | Additional comments | String |

**Appendix 3. Summary table of studies included**

| **Event number** | **Author** | **Journal** | **Disease** | **Country** | **Year of transmission** | **Year of publication** | **Delay to publication (years)** | **Setting** | **Transmission pathway** | **Number of cases minus source** | **Number of deaths** | **Type of study** |
| --- | --- | --- | --- | --- | --- | --- | --- | --- | --- | --- | --- | --- |
| 1 | [Heikens E](https://pubmed.ncbi.nlm.nih.gov/?term=Heikens+E&cauthor_id=30500385) | J Hosp Infect | C | Netherlands | 2016 | 2018 | 2 | Dialysis unit | Haemodialysis machine | 2 | 1 - non hep | Study reporting phylogenetic analysis |
| 2 | Halleck F | Transpl. Int | C | Germany | Not specified | 2017 |  | Surgery | Organ donor | 5 | 1 - non hep | Study not reporting phylogenetic analysis |
| 3 | [McDermott J](https://pubmed.ncbi.nlm.nih.gov/?term=McDermott+J&cauthor_id=30633945) | J Hosp Infect | C | Italy | 2005 | 2019 | 14 | Haematology/oncology unit | Unspecified IPC breach | 5 | 1 - not specified | Study reporting phylogenetic analysis |
| 4 | Caraballo K | PLoS One | C | Poland | 2015 | 2018 | 3 | Haematology/oncology unit | Unspecified IPC breach | 7 | 4 - non hep | Study reporting phylogenetic analysis |
| 5 | [Garvey M](https://pubmed.ncbi.nlm.nih.gov/?term=Garvey+MI&cauthor_id=28196726) | J Hosp Infect | C | United Kingdom | Not specified | 2017 |  | Dialysis unit | Unspecified IPC breach | 1 | 0 | Study reporting phylogenetic analysis |
| 6 | [Johannessen I](https://pubmed.ncbi.nlm.nih.gov/?term=Johannessen+I&cauthor_id=29242141) | J Hosp Infect | C | United Kingdom | 2013 | 2017 | 4 | Emergency department | Unspecified IPC breach | 1 | 0 | Study reporting phylogenetic analysis |
| 7 | [Candotti D](https://pubmed.ncbi.nlm.nih.gov/?term=Candotti+D&cauthor_id=29959168) | Gut | B | Slovenia | 2008 | 2018 | 10 | Multi settings blood products | Blood product | 7 | 0 | Lookback investigation |
| 7 | [Candotti D](https://pubmed.ncbi.nlm.nih.gov/?term=Candotti+D&cauthor_id=29959168) | Gut | B | Slovenia | 2008 | 2018 | 10 | Multi settings blood products | Blood product | 1 | 0 | Lookback investigation |
| 7 | [Candotti D](https://pubmed.ncbi.nlm.nih.gov/?term=Candotti+D&cauthor_id=29959168) | Gut | B | Slovenia | 2008 | 2018 | 10 | Multi settings blood products | Blood product | 1 | 0 | Lookback investigation |
| 8 | [Mazzucco W](https://pubmed.ncbi.nlm.nih.gov/?term=Mazzucco+W&cauthor_id=34171407) | J Hosp Infect | C | Italy | 2016 | 2021 | 5 | Outpatients | Blood product | 2 | 0 | Study reporting phylogenetic analysis |
| 8 | Mazzucco W | J Hosp Infect | C | Italy | 2016 | 2021 | 5 | Outpatients | Blood product | 1 | 0 | Study reporting phylogenetic analysis |
| 8 | Mazzucco W | J Hosp Infect | C | Italy | 2016 | 2021 | 5 | Outpatients | Blood product | 1 | 0 | Study reporting phylogenetic analysis |
| 8 | Mazzucco W | J Hosp Infect | C | Italy | 2016 | 2021 | 5 | Outpatients | Blood product | 3 | 0 | Study reporting phylogenetic analysis |
| 9 | [Brancaccio G](https://www.researchgate.net/profile/Giuseppina-Brancaccio) | [J. Hepatol](https://www.researchgate.net/journal/Journal-of-Hepatology-0168-8278) | C | Italy | 2016 | 2017 | 1 | Haematology/oncology unit | Unidentified | 4 | 3 - non hep | Study reporting phylogenetic analysis |
| 10 | [Zakrzewska K](https://pubmed.ncbi.nlm.nih.gov/?term=Zakrzewska+K&cauthor_id=31385675) | Przegl Epidemiol | C | Poland | 2016 | 2019 | 3 | CT/MRI scanning unit | Contrast media injector | 8 | 0 | Study not reporting phylogenetic analysis |
| 11 | [Spreafico M](https://www.researchgate.net/profile/Marta-Spreafico) | [Dig Liver Dis](https://www.researchgate.net/journal/Digestive-and-Liver-Disease-1590-8658) | B | Italy | Not specified | 2012 |  | Multi settings blood products | Blood product | 1 | 0 | Study reporting phylogenetic analysis |
| 12 | [Lieshout-Krikke R](https://pubmed.ncbi.nlm.nih.gov/?term=Lieshout-Krikke+RW&cauthor_id=26559665) | Transfusion | B | Netherlands | 2007 | 2016 | 9 | Multi settings blood products | Blood product | 2 | 0 | Lookback investigation |
| 13 | [Stępień M](https://pubmed.ncbi.nlm.nih.gov/?term=St%C4%99pie%C5%84+M&cauthor_id=26519841) | Przegl Epidemiol | C | Poland | 2006 | 2015 | 9 | Dialysis unit | Unspecified IPC breach | 53 | 0 | Study reporting phylogenetic analysis |
| 13 | Stępień M | Przegl Epidemiol | C | Poland | 2011 | 2015 | 4 | Dialysis unit | Unidentified | 5 | 0 | Study reporting phylogenetic analysis |
| 13 | Stępień M | Przegl Epidemiol | C | Poland | 2013 | 2015 | 2 | Haematology/oncology unit | Unidentified | 8 | 0 | Study reporting phylogenetic analysis |
| 13 | Stępień M | Przegl Epidemiol | C | Poland | 2013 | 2015 | 2 | CT/MRI scanning unit | Contrast media injector | 9 | 0 | Study reporting phylogenetic analysis |
| 14 | [Spreafico M](https://pubmed.ncbi.nlm.nih.gov/?term=Spreafico+M&cauthor_id=26116791) | J. Hepatol | B | Italy | 2007 | 2015 | 8 | Multi settings blood products | Blood product | 1 | 0 | Lookback investigation |
| 14 | Spreafico M | J. Hepatol | B | Italy | 2007 | 2015 | 8 | Multi settings blood products | Blood product | 1 | 0 | Lookback investigation |
| 15 | [Kliner M](https://pubmed.ncbi.nlm.nih.gov/?term=Kliner+M&cauthor_id=25713718) | Clin. Kidney J | B | United Kingdom | 2011 | 2015 | 4 | Inpatient ward | Unspecified IPC breach | 1 | 0 | Study not reporting phylogenetic analysis |
| 16 | [Diercke M](https://pubmed.ncbi.nlm.nih.gov/?term=Diercke+M&cauthor_id=25611818) | J Med Virol | B | Germany | 2010 | 2015 | 5 | Nursing home | Capillary blood sampling | 5 | 0 | Study reporting phylogenetic analysis |
| 17 | [Pekova L](https://pubmed.ncbi.nlm.nih.gov/?term=Pekova+LM&cauthor_id=17719679) | J Hosp Infect | C | Bulgaria | 2004 | 2007 | 3 | Inpatient ward | Multi-dose vial contamination | 6 | 1 - non hep | Study not reporting phylogenetic analysis |
| 18 | [Webster D](https://www.researchgate.net/profile/Daniel-Webster) | J Hosp Infect | C | United Kingdom | 2000 | 2007 | 7 | Dialysis unit | Haemodialysis machine | 1 | 0 | Study reporting phylogenetic analysis |
| 19 | [Kretzschmar E](https://pubmed.ncbi.nlm.nih.gov/?term=Kretzschmar+E&cauthor_id=17456153) | Vox Sang | C | Germany | 2004 | 2007 | 3 | Multi settings blood products | Blood product | 1 | 0 | Lookback investigation |
| 20 | [Kondili L](https://pubmed.ncbi.nlm.nih.gov/?term=Kondili+LA&cauthor_id=16835740) | Eur J Clin Microbiol Infect Dis | B | Italy | 2003 | 2006 | 3 | Dialysis unit | Unspecified IPC breach | 2 | 0 | Study reporting phylogenetic analysis |
| 20 | [Kondili L](https://pubmed.ncbi.nlm.nih.gov/?term=Kondili+LA&cauthor_id=16835740) | Eur J Clin Microbiol Infect Dis | C | Italy | 2003 | 2006 | 3 | Dialysis unit | Unidentified | 4 | 0 | Study reporting phylogenetic analysis |
| 21 | [Bracho M](https://pubmed.ncbi.nlm.nih.gov/?term=Bracho+MA&cauthor_id=16597853) | J Clin Microbiol | B | Spain | 2002 | 2006 | 4 | Haematology/oncology unit | Unidentified | 2 | 0 | Study reporting phylogenetic analysis |
| 22 | [Dreesman J](https://www.ncbi.nlm.nih.gov/pubmed/?term=DREESMAN%20J%5BAuthor%5D&cauthor=true&cauthor_uid=16438748) | [Epidemiol Infect.](https://www.ncbi.nlm.nih.gov/pmc/articles/PMC2870482/) | B | Germany | 2001 | 2006 | 5 | Nursing home | Capillary blood sampling | 16 | 0 | Study reporting phylogenetic analysis |
| 23 | [Stark K](https://pubmed.ncbi.nlm.nih.gov/?term=Stark+K&cauthor_id=16329003) | Arch Virol | C | Germany | 2001 | 2006 | 5 | Surgery | Unspecified IPC breach | 3 | 0 | Study reporting phylogenetic analysis |
| 24 | [Pourkarim M](https://pubmed.ncbi.nlm.nih.gov/?term=Pourkarim+MR&cauthor_id=19615936) | J Clin Virol | B | Belgium | 2003 | 2009 | 6 | Primary care | Unspecified IPC breach | 32 | 0 | Study reporting phylogenetic analysis |
| 25 | [Ross R](https://pubmed.ncbi.nlm.nih.gov/?term=Ross+RS&cauthor_id=19107970) | J Med Virol | C | Germany | 2001 | 2009 | 8 | Inpatient ward | Unidentified | 5 | 0 | Study reporting phylogenetic analysis |
| 26 | [Rorat M](https://pubmed.ncbi.nlm.nih.gov/?term=Rorat+M&cauthor_id=24388479) | Am J Infect Control | C | Poland | 2001 | 2014 | 13 | Inpatient ward | Unspecified IPC breach | 26 | 0 | Study not reporting phylogenetic analysis |
| 27 | [Duffell E](https://pubmed.ncbi.nlm.nih.gov/?term=Duffell+EF&cauthor_id=20478083) | Epidemiol Infect | B | United Kingdom | 2004 | 2011 | 7 | Nursing home | Capillary blood sampling | 6 | 2 - not specified | Study reporting phylogenetic analysis |
| 27 | [Duffell E](https://pubmed.ncbi.nlm.nih.gov/?term=Duffell+EF&cauthor_id=20478083) | Epidemiol Infect | B | United Kingdom | 2004 | 2011 | 7 | Nursing home | Capillary blood sampling | 1 | 0 | Study reporting phylogenetic analysis |
| 27 | Duffell E | Epidemiol Infect | B | United Kingdom | 2004 | 2011 | 7 | Nursing home | Capillary blood sampling | 5 | 2 - not specified | Study reporting phylogenetic analysis |
| 27 | Duffell E | Epidemiol Infect | B | United Kingdom | 2004 | 2011 | 7 | Nursing home | Capillary blood sampling | 1 | 1 - not specified | Study reporting phylogenetic analysis |
| 27 | Duffell E | Epidemiol Infect | B | United Kingdom | 2004 | 2011 | 7 | Nursing home | Capillary blood sampling | 6 | 1 - not specified | Study reporting phylogenetic analysis |
| 28 | [Thomson P](https://www.ncbi.nlm.nih.gov/pubmed/?term=Thomson%20PC%5BAuthor%5D&cauthor=true&cauthor_uid=25984097) | [NDT Plus.](https://www.ncbi.nlm.nih.gov/pmc/articles/PMC4421637/) | C | United Kingdom | 2009 | 2011 | 2 | Dialysis unit | Haemodialysis machine | 1 | 0 | Study reporting phylogenetic analysis |
| 29 | [Olsen K](https://pubmed.ncbi.nlm.nih.gov/?term=Olsen+K&cauthor_id=20971233) | Ann Thorac Surg | C | Norway | 2005 | 2010 | 5 | Surgery | Unspecified IPC breach | 10 | 0 | Study reporting phylogenetic analysis |
| 30 | [Almroth G](https://pubmed.ncbi.nlm.nih.gov/?term=Almroth+G&cauthor_id=20029812) | J Med Virol | C | Sweden | 2001 | 2010 | 9 | Dialysis unit | Unidentified | 1 | 0 | Study reporting phylogenetic analysis |
| 31 | [Rodríguez-Caravaca G](https://pubmed.ncbi.nlm.nih.gov/?term=Rodr%C3%ADguez-Caravaca+G&cauthor_id=19646793) | Enferm Infecc Microbiol Clin | C | Spain | 2003 | 2010 | 7 | Haematology/oncology unit | Multi-dose vial contamination | 7 | 0 | Study not reporting phylogenetic analysis |
| 32 | [Spada E](https://pubmed.ncbi.nlm.nih.gov/?term=Spada+E&cauthor_id=18098132) | J Med Virol | C | Italy | 2003 | 2008 | 5 | Dialysis unit | Unspecified IPC breach | 4 | 0 | Study reporting phylogenetic analysis |
| 33 | [Girou E](javascript:;) | Clin Infect Dis | C | France | 2004 | 2008 | 4 | Dialysis unit | Unspecified IPC breach | 1 | 0 | Study reporting phylogenetic analysis |
| 34 | [Pañella H](https://www.ncbi.nlm.nih.gov/pubmed/?term=Pa%26%23x000f1%3Bella%20H%5BAuthor%5D&cauthor=true&cauthor_uid=18258135) | [Emerg Infect Dis](https://www.ncbi.nlm.nih.gov/pmc/articles/PMC2600211/) | C | Spain | 2004 | 2008 | 4 | CT/MRI scanning unit | Unidentified | 3 | 0 | Study reporting phylogenetic analysis |
| 34 | Pañella H | [Emerg Infect Dis](https://www.ncbi.nlm.nih.gov/pmc/articles/PMC2600211/) | C | Spain | 2004 | 2008 | 4 | CT/MRI scanning unit | Unidentified | 1 | 0 | Study reporting phylogenetic analysis |
| 34 | Pañella H | [Emerg Infect Dis](https://www.ncbi.nlm.nih.gov/pmc/articles/PMC2600211/) | C | Spain | 2004 | 2008 | 4 | CT/MRI scanning unit | Unidentified | 1 | 0 | Study reporting phylogenetic analysis |
| 34 | Pañella H | [Emerg Infect Dis](https://www.ncbi.nlm.nih.gov/pmc/articles/PMC2600211/) | C | Spain | 2004 | 2008 | 4 | Dialysis unit | Unidentified | 1 | 0 | Study reporting phylogenetic analysis |
| 35 | [Poujol I](https://pubmed.ncbi.nlm.nih.gov/?term=Poujol+I&cauthor_id=18761965) | Euro Surveill | B | France | 2005 | 2008 | 3 | Surgery | Unspecified IPC breach | 1 | 0 | Study not reporting phylogenetic analysis |
| 36 | [Levicnik-Stezinar S](https://pubmed.ncbi.nlm.nih.gov/?term=Levicnik-Stezinar+S&cauthor_id=18436328) | J Hepatol | B | Slovenia | 2005 | 2008 | 3 | Multi settings blood products | Blood product | 2 | 0 | Lookback investigation |
| 37 | [Burns K](https://pubmed.ncbi.nlm.nih.gov/?term=Burns+K&cauthor_id=21530000) | J Hosp Infect | B | Ireland | 2005 | 2011 | 6 | Inpatient ward | Unspecified IPC breach | 1 | 0 | Study reporting phylogenetic analysis |
| 37 | Burns K | J Hosp Infect | B | Ireland | 2005 | 2011 | 6 | Inpatient ward | Unspecified IPC breach | 1 | 0 | Study reporting phylogenetic analysis |
| 37 | Burns K | J Hosp Infect | B | Ireland | 2005 | 2011 | 6 | Inpatient ward | Unspecified IPC breach | 1 | 0 | Study reporting phylogenetic analysis |
| 37 | Burns K | J Hosp Infect | B | Ireland | 2005 | 2011 | 6 | Inpatient ward | Unspecified IPC breach | 1 | 0 | Study reporting phylogenetic analysis |
| 38 | [Dencs A](https://pubmed.ncbi.nlm.nih.gov/?term=Dencs+A&cauthor_id=21264863) | J Med Virol | C | Hungary | 2007 | 2011 | 4 | Haematology/oncology unit | Unidentified | 20 | 0 | Study reporting phylogenetic analysis |
| 39 | [Muir D](https://pubmed.ncbi.nlm.nih.gov/?term=Muir+D&cauthor_id=24166559) | J Med Virol | C | United Kingdom | 2011 | 2014 | 3 | Inpatient ward | Unidentified | 1 | 0 | Study reporting phylogenetic analysis |
| 40 | [Servant-Delmas A](https://pubmed.ncbi.nlm.nih.gov/?term=Servant-Delmas+A&cauthor_id=22671296) | Transfusion | B | France | 2007 | 2012 | 5 | Multi settings blood products | Blood product | 1 | 0 | Lookback investigation |
| 40 | Servant-Delmas A | Transfusion | B | France | 2007 | 2012 | 5 | Multi settings blood products | Blood product | 1 | 0 | Lookback investigation |
| 41 | [Lieshout-Krikke R](https://pubmed.ncbi.nlm.nih.gov/?term=Lieshout-Krikke+RW&cauthor_id=21883273) | Vox Sang | B | Netherlands | 2006 | 2012 | 6 | Multi settings blood products | Blood product | 2 | 0 | Lookback investigation |
| 42 | [Coppola N](https://pubmed.ncbi.nlm.nih.gov/?term=Coppola+N&cauthor_id=23856167) | J Clin Virol | B | Italy | 2011 | 2013 | 2 | Multi settings blood products | Blood product | 1 | 0 | Lookback investigation |
| 43 | [Saludes V](https://pubmed.ncbi.nlm.nih.gov/?term=Saludes+V&cauthor_id=23567025) | J Clin Virol | C | Spain | 2010 | 2013 | 3 | Outpatients | Multi-dose vial contamination | 1 | 0 | Study reporting phylogenetic analysis |
| 44 | [Torsvik Steinsvåg C](https://pubmed.ncbi.nlm.nih.gov/?term=Steinsv%C3%A5g+CT&cauthor_id=24157220) | Transfus Apher Sci | C | Norway | 2010 | 2013 | 3 | Multi settings blood products | Blood product | 1 | 0 | Study not reporting phylogenetic analysis |
| 45 | [Senatore S](https://pubmed.ncbi.nlm.nih.gov/?term=Senatore+S&cauthor_id=27613441) | J Hosp Infect | C | Italy | 2013 | 2016 | 3 | Dialysis unit | Unspecified IPC breach | 3 | 0 | Study reporting phylogenetic analysis |
| 46 | [Kološová A](https://pubmed.ncbi.nlm.nih.gov/?term=Kolo%C5%A1ov%C3%A1+A&cauthor_id=26944899) | J Hosp Infect | B | Slovakia | 2010 | 2016 | 6 | Haematology/oncology unit | Unidentified | 29 | 0 | Study not reporting phylogenetic analysis |
| 46 | Kološová A | J Hosp Infect | C | Slovakia | 2010 | 2016 | 6 | CT/MRI scanning unit | Contrast media injector | 14 | 0 | Study not reporting phylogenetic analysis |
| 47 | [Aho-Glélé L](https://pubmed.ncbi.nlm.nih.gov/?term=Aho-Gl%C3%A9l%C3%A9+LS&cauthor_id=26510471) | Infect Control Hosp Epidemiol | C | France | 2013 | 2016 | 3 | Dialysis unit | Unspecified IPC breach | 1 | 0 | Study reporting phylogenetic analysis |
| 48 | [Donohue S](https://pubmed.ncbi.nlm.nih.gov/?term=Donohue+S&cauthor_id=22269250) | J Hosp Infect | C | Ireland | 2000 | 2012 | 12 | Unidentified | Unidentified | 1 | 0 | Study not reporting phylogenetic analysis |
| 49 | [Bourigault C](https://www.journalofhospitalinfection.com/article/S0195-6701(11)00265-9/fulltext) | J Hosp Infect | C | France | 2008 | 2011 | 3 | Home nursing care | Unspecified IPC breach | 1 | 0 | Study reporting phylogenetic analysis |
| 50 | [Laurenson I](https://pubmed.ncbi.nlm.nih.gov/?term=Laurenson+IF&cauthor_id=17602792) | J Hosp Infect | B | United Kingdom | 2001 | 2007 | 6 | Surgery | Unidentified | 3 | 1 - not specified | Study not reporting phylogenetic analysis |
| 51 | [Manuel F](https://www-aerzteblatt-de.translate.goog/archiv/suche?autor=Furtw%EF%BF%BDngler,+Manuel&_x_tr_sl=de&_x_tr_tl=en&_x_tr_hl=sv&_x_tr_pto=nui) | [Dtsch Ärzteebl](https://www-aerzteblatt-de.translate.goog/archiv/inhalt?heftid=2646&_x_tr_sl=de&_x_tr_tl=en&_x_tr_hl=sv&_x_tr_pto=nui) | B | Germany | Not specified | 2006 |  | Surgery | Unspecified IPC breach | 2 | 0 | Study not reporting phylogenetic analysis |
| 51 | Manuel F | [Dtsch Ärzteebl](https://www-aerzteblatt-de.translate.goog/archiv/inhalt?heftid=2646&_x_tr_sl=de&_x_tr_tl=en&_x_tr_hl=sv&_x_tr_pto=nui) | B | Germany | Not specified | 2006 |  | Surgery | Unspecified IPC breach | 1 | 0 | Study not reporting phylogenetic analysis |
| 51 | Manuel F | [Dtsch Ärzteebl](https://www-aerzteblatt-de.translate.goog/archiv/inhalt?heftid=2646&_x_tr_sl=de&_x_tr_tl=en&_x_tr_hl=sv&_x_tr_pto=nui) | B | Germany | Not specified | 2006 |  | Inpatient ward | Blood product | 1 | 0 | Study not reporting phylogenetic analysis |
| 52 | [Forns X](https://pubmed.ncbi.nlm.nih.gov/?term=Forns+X&cauthor_id=15619236) | Hepatology | C | Spain | 2000 | 2008 | 8 | Inpatient ward | Unspecified IPC breach | 4 | 0 | Study reporting phylogenetic analysis |
| 53 | [Gotz H](https://www.eurosurveillance.org/search?value1=H+M+Gotz&option1=author&noRedirect=true) | Euro Surveill | B | Netherlands | 2007 | 2007 | 0 | Nursing home | Capillary blood sampling | 2 | 1 - non hep | Study not reporting phylogenetic analysis |
| 54 | [González-Candelas F](https://virologyj.biomedcentral.com/articles/10.1186/1743-422X-7-217#auth-Fernando-Gonz_lez_Candelas) | [Virol J](https://virologyj.biomedcentral.com/) | C | Spain | 2005 | 2010 | 5 | Outpatients | Unspecified IPC breach | 2 | 0 | Study reporting phylogenetic analysis |
| 55 | [Lanini S](https://bmcinfectdis.biomedcentral.com/articles/10.1186/1471-2334-10-257#auth-Simone-Lanini) | [BMC Infect. Dis](https://bmcinfectdis.biomedcentral.com/) | C | Italy | 2004 | 2010 | 6 | Dialysis unit | Unspecified IPC breach | 11 | 0 | Study reporting phylogenetic analysis |
| 55 | Lanini S | BMC Infect. Dis | C | Italy | 2004 | 2010 | 6 | Dialysis unit | Unidentified | 2 | 0 | Study reporting phylogenetic analysis |
| 56 | [Dencs A](https://pubmed.ncbi.nlm.nih.gov/?term=Dencs+A&cauthor_id=21450552) | Acta Microbiol Immunol Hung | B | Hungary | 2008 | 2011 | 3 | Haematology/oncology unit | Unspecified IPC breach | 8 | 0 | Study not reporting phylogenetic analysis |
| 57 | [Lanini S](https://pubmed.ncbi.nlm.nih.gov/?term=Lanini+S&cauthor_id=22412991) | PLoS One | B | Italy | 2006 | 2012 | 6 | Haematology/oncology unit | Capillary blood sampling | 3 | 0 | Study not reporting phylogenetic analysis |
| 58 | Varnier O | ESCMID | C | Italy | 2005 | Not specified |  | Inpatient ward | Multi-dose vial contamination | 5 | 1 - not specified | Study reporting phylogenetic analysis |
| 59 | Gerlich W | Vox Sanguinis | B | Germany | Not specified | Not specified |  | Multi settings blood products | Blood product | 3 | 3 - fulminant hepatitis | Lookback investigation |
| 60 | [Spreafico M](https://www.researchgate.net/profile/Marta-Spreafico) | [Dig Liver Dis](https://www.researchgate.net/journal/Digestive-and-Liver-Disease-1590-8658) | B | Italy | Not specified | 2012 |  | Multi settings blood products | Blood product | 1 | 0 | Lookback investigation |
| 61 | [Seiz P](https://pubmed.ncbi.nlm.nih.gov/?term=Seiz+PL&cauthor_id=26338147) | Int J Med Microbiol | B | Germany | Not specified | 2015 |  | Nursing home | Unidentified | 14 | 2 - fulminant hepatitis | Study reporting phylogenetic analysis |
| 61 | Seiz P | Int J Med Microbiol | B | Germany | Not specified | 2015 |  | Nursing home | Unidentified | 6 | 0 | Study reporting phylogenetic analysis |
| 62 | Erikstrup C | Vox Sanguinis | B | France | Not specified | 2011 |  | Multi settings blood products | Blood product | 2 | 0 | Lookback investigation |
| 63 | Mayerhofer T | ISMD2014 | B | Austria | Not specified | 2014 |  | Multi settings blood products | Blood product | 1 | 0 | Lookback investigation |
| 64 | van Kraaij M | Transfusion | B | Netherlands | Not specified | 2012 |  | Multi settings blood products | Blood product | 4 | 0 | Lookback investigation |
| 65 | Nassauer A | Bundesgesundheitsbl | C | Germany | 2006 | 2009 | 3 | Inpatient ward | Unidentified | 1 | 0 | Study not reporting phylogenetic analysis |

*Note:* IPC - infection prevention and control, CT – computed tomography, MRI – magnetic resonance imaging
